# Novel Systemic Inflammation Markers to Predict COVID-19 Prognosis

**DOI:** 10.3389/fimmu.2021.741061

**Published:** 2021-10-22

**Authors:** Amirali Karimi, Parnian Shobeiri, Arutha Kulasinghe, Nima Rezaei

**Affiliations:** ^1^School of Medicine, Tehran University of Medical Sciences, Tehran, Iran; ^2^Network of Immunity in Infection, Malignancy and Autoimmunity (NIIMA), Universal Scientific Education and Research Network (USERN), Tehran, Iran; ^3^Research Center for Immunodeficiencies, Pediatrics Center of Excellence, Children’s Medical Center, Tehran University of Medical Sciences, Tehran, Iran; ^4^Non-Communicable Diseases Research Center, Endocrinology and Metabolism Population Sciences Institute, Tehran University of Medical Sciences, Tehran, Iran; ^5^Centre for Genomics and Personalised Health, School of Biomedical Q6 Sciences, Queensland University of Technology, Brisbane, QL, Australia; ^6^Department of Immunology, School of Medicine, Tehran University of Medical Sciences, Tehran, Iran

**Keywords:** biomarkers, COVID-19, inflammation, inflammatory markers, prognosis, SARS-CoV-2

## Abstract

Coronavirus disease 2019 (COVID-19) has resulted in a global pandemic, challenging both the medical and scientific community for the development of novel vaccines and a greater understanding of the effects of the SARS-CoV-2 virus. COVID-19 has been associated with a pronounced and out-of-control inflammatory response. Studies have sought to understand the effects of inflammatory response markers to prognosticate the disease. Herein, we aimed to review the evidence of 11 groups of systemic inflammatory markers for risk-stratifying patients and prognosticating outcomes related to COVID-19. Numerous studies have demonstrated the effectiveness of neutrophil to lymphocyte ratio (NLR) in prognosticating patient outcomes, including but not limited to severe disease, hospitalization, intensive care unit (ICU) admission, intubation, and death. A few markers outperformed NLR in predicting outcomes, including 1) systemic immune-inflammation index (SII), 2) prognostic nutritional index (PNI), 3) C-reactive protein (CRP) to albumin ratio (CAR) and high-sensitivity CAR (hsCAR), and 4) CRP to prealbumin ratio (CPAR) and high-sensitivity CPAR (hsCPAR). However, there are a limited number of studies comparing NLR with these markers, and such conclusions require larger validation studies. Overall, the evidence suggests that most of the studied markers are able to predict COVID-19 prognosis, however NLR seems to be the most robust marker.

## 1 Introduction

Coronavirus disease 2019 (COVID-19) has emerged as a global challenge of the modern healthcare systems, resulting in more than 177 million confirmed cases and nearly 4 million deaths (1–3). Severe acute respiratory syndrome-coronavirus 2 (SARS-CoV-2) infection can involve various organs and produce a wide range of symptoms (4–9). Multiple organ involvement is thought to occur due to the almost universal distribution of angiotensin-converting enzyme 2 (ACE-2) that attaches to SARS-CoV-2 spike (S) protein receptor binding domain (RBD) and type 2 transmembrane serine proteases (TMPRSS2) that cleaves the S protein. It is thought that both these molecules may initiate immune evasion through various mechanisms (10–13).

It is well-documented that inflammatory mechanisms play a principal role in COVID-19-related organ dysfunction and mortality (14, 15). Patients with COVID-19 typically have higher inflammatory cytokines such as IL-6 and TNF-α compared with healthy individuals (16). Furthermore, patients with COVID-19 experience elevated levels of serologic indicators of inflammation, such as C-reactive protein (CRP), erythrocyte sedimentation rate (ESR), lactate dehydrogenase (LDH), and procalcitonin (17, 18). These inflammatory cytokines may also alter the levels of various blood cell lineages and notably cause lymphocytopenia (18, 19). This hyperinflammation plays an important role in viral pathogenesis. However, it is also possible to use this proinflammatory response to risk-stratify COVID-19 patients at high risk of developing severe disease and respiratory complications (20).

Historically, markers of inflammation were used to successfully prognosticate patients with inflammatory diseases and, in particular, various types of cancers (21–25). Previous studies examined the role of inflammatory markers in other infectious diseases and demonstrated their ability to risk-stratify patients (26). Herein, we aimed to review the evidence for the effectiveness of systemic inflammatory markers in risk-stratifying patients and prognosticating outcomes related to COVID-19. The markers include neutrophil to lymphocyte ratio (NLR) and derived NLR (d-NLR), platelet to lymphocyte ratio (PLR), lymphocyte to monocyte ratio (LMR), lymphocyte to CRP ratio (LCR), fibrinogen to prealbumin ratio (FPR) and albumin to fibrinogen ratio (AFR), CRP to albumin ratio (CAR) and CRP to prealbumin ratio (CPAR), Glasgow prognostic score (GPS), modified GPS (mGPS), high-sensitivity mGPS (HS-mGPS), prognostic index (PI), prognostic nutritional index (PNI), systemic immune-inflammation index (SII), and interferon-alpha-inducible protein 27 (IFI27).

## 2 Inflammatory Markers

### 2.1 Neutrophil to Lymphocyte Ratio and Derived NLR

NLR is defined as the absolute neutrophil count (ANC)/absolute lymphocyte count (ALC) (27–29). d-NLR has a similar definition to NLR, calculated as ANC/(White blood cells (WBC) total count − ANC) (30). If we consider monocyte, basophil, and eosinophil levels as negligible (which are mostly not), the definition of these two markers would be equal. Earlier studies found links to higher NLR or d-NLR in chronic conditions with low-grade inflammatory nature, such as obesity, hypertension, diabetes mellitus, metabolic syndrome, atherosclerotic events of the heart and brain, and various cancers, although previous literature studied NLR more than d-NLR (27–29, 31–33). These underlying diseases are considered as risk factors for severe COVID-19 (2, 34, 35).

Since the beginning of the pandemic, studies have investigated the role of NLR in COVID-19 prognostication and its utility as a biomarker. NLR has been reported to prognosticate mortality, progression to severe disease, risk of intubation, risk of severe disease in intubated patients, days intubated, ICU admission, and longer intensive care unit (ICU) admission (30, 36–57). Two meta-analyses of *n* = 19 and *n* = 13 studies found significant associations between higher NLR and COVID-19 severity and mortality (58, 59). Furthermore, patients with higher NLR appear to have more comorbidities and, therefore, are more prone to severe COVID-19 (36). Even in patients with comorbidities, NLR might maintain its predictive ability for COVID-19 severity. For instance, NLR significantly predicted COVID-19 severity and survival in hospitalized patients with different types of cancers (60, 61). It has been suggested that each increased NLR unit resulted in an 8% higher mortality in COVID-19 patients (45).

A temporal analysis showed that on-admission NLR correlates well with the need for ICU and poor outcomes, and can be a potential risk-stratification tool. However, the clinical utility of NLR was lost in week 3 post-admission (62). The best predictive value of NLR can be achieved at its peak compared with its on-admission values (63). Concurrently, another study on the temporality of NLR found that day 7 measurement of NLR could significantly predict those requiring invasive mechanical ventilation and mortality, while measurement of day 1 NLR could not (64). In summary, on-admission NLR could predict COVID-19 prognosis. This predictive ability increases for a few days after admission, when NLR reaches its peak. However, NLR gradually loses its predictive ability as the patient recovers from COVID-19 and an associated reduction in inflammation. Finally, at week 3 post-admission, NLR loses its clinical utility to prognosticate severe COVID-19 outcomes.

Five studies proposed the ability of NLR to assist COVID-19 diagnosis (49, 65–68). They defined assisting COVID-19 diagnosis as significantly higher levels of NLR in individuals with COVID-19 compared with healthy controls. However, none of the studies mentioned how and due to what situations NLR can be integrated into COVID-19 diagnosis. Two other markers have been reported to be predictive for COVID-19 disease severity and mortality: granulocyte to lymphocyte ratio (69) and d-NLR (30, 70, 71).

#### 2.1.1 NLR in Comparison With Other Markers

In **Tables 1**, **2**, we compared NLR to other reported markers for COVID-19. We seperated the variables into those that have been reported for COVID-19 diagnosis and disease severity. **Table 1** summarizes the studies comparing NLR to only LMR, PLR, and d-NLR. NLR had the highest predictive value compared with LMR, PLR, and d-NLR in most of the studies for severe COVID-19 parameters—disease severity, ICU admission, progression to acute respiratory distress syndrome (ARDS), need for mechanical ventilation, duration and expense of hospital stay, time to negative PCR, and mortality.

**Table 1:** Studies comparing NLR to only PLR, LMR, and d-NLR among different measured variables.

Study	Measured variable	Summary of findings
(49)	COVID-19 diagnosis	In intubated COVID-19 patients: higher NLR and PLR and lower LMR were observed compared with healthy individuals.
(65)	COVID-19 diagnosis	NLR, PLR, and MLR were all higher in COVID-19 patients [order of higher AUC: MLR (0.892) > PLR (0.748) > NLR (0.722)].
(66)	COVID-19 diagnosis	Both NLR and PLR correlated (order of higher correlation: NLR > PLR).
(67)	COVID-19 diagnosis	Both NLR and PLR were higher is SARS-CoV-2 (+) patients [order of higher AUC: PLR (0.669) > NLR (0.615)].
(30)	COVID-19 pneumonia	NLR correlated in the multivariate analysis, but d-NLR, LMR, and PLR did not.
(30)	Disease severity	NLR, d-NLR, and PLR correlated with disease severity (order of better prediction: NLR > d-NLR > PLR). LMR did not correlate.
(59)	Disease severity	In this meta-analysis, NLR correlated better than PLR (SMD: 2.80 vs. 1.82).
(72)	Disease severity	In patients with type 2 diabetes mellitus: both NLR and LMR correlated with disease severity (AUC: NLR = 0.730, *p* = 0.002; LMR = 0.322, *p* = 0.015).
(73)	Disease severity	NLR, d-NLR, and PLR correlated with disease severity [order of higher AUC: NLR (0.808) > d-NLR (0.803) > PLR (0.769)]. LMR did not correlate (AUC = 0.296).
(74)	Disease severity	NLR remained independently related in the logistic regression analysis. PLR only correlated in the univariate analysis. No correlation was observed for LMR.
(53)	Disease severity	NLR, PLR, and LMR could predict disease severity (order of higher AUC: NLR > LMR > PLR).
(65)	Progression to ARDS	NLR, PLR, and LMR predicted progression to ARDS.
(53)	ICU admission	Among patients with severe disease, NLR correlated with ICU admission, but LMR and PLR did not.
(75)	ICU admission	NLR, PLR, and LMR predicted ICU admission (order of better prediction: NLR > PLR > LMR).
(76)	Mechanical ventilation	NLR predicted the need for mechanical ventilation, but PLR did not.
(72)	Time to negative PCR	In patients with type 2 diabetes mellitus, NLR correlated (multivariate analysis), but LMR did not (univariate analysis).
(72)	Duration of hospital stay	In patients with type 2 diabetes mellitus, NLR independently correlated but LMR was not related in the univariate analysis.
(77)	In-hospital mortality	NLR and d-NLR correlated, but LMR and PLR did not.
(66)	Mortality	NLR correlated, but PLR did not.
(78)	All-cause mortality	NLR could predict this parameter, but PLR could not.
(72)	Hospital expenses	In patients with type 2 diabetes mellitus, NLR independently correlated but LMR did not correlate in the univariate analysis.

NLR, neutrophil to lymphocyte ratio; PLR, platelet to lymphocyte ratio; LMR, lymphocyte to monocyte ratio; MLR, monocyte to lymphocyte ratio; d-NLR, derived-NLR; AUC, area under the curve; SMD, standardized mean difference; ARDS, acute respiratory distress syndrome; ICU, intensive care unit; PCR, polymerase chain reaction.

**Table 2:** Studies comparing NLR with other biomarkers (studies involving discussed markers other than PLR, LMR, and d-NLR) among different measured variables.

Study	Measured variable	Summary of findings
(68)	COVID-19 diagnosis	SII and NLR were higher in patients with SARS-CoV-2 diagnosis in the multivariate analysis (order of higher AUC: SII > NLR). PLR did not correlate.
(71)	Disease severity	Higher hsCAR, higher hsCPAR, and lower PNI correlated in the multivariate analysis, but d-NLR and SII only correlated in the univariate analysis. NLR, PLR, LMR, and AFR did not correlate.
(79)	Disease severity	Both CAR and NLR predicted disease severity in the multivariate analysis, but CAR had higher OR (OR = 17.65, *p* = 0.001 vs. OR = 1.51, *p* = 0.007).
(80)	Disease severity	In this meta-analysis, both NLR and LCR predicted disease severity [order of better prediction: NLR (SMD: 2.404) > LCR (SMD: −0.912)]
(61)	Mortality	In cancer patients: higher NLR, lower PNI, higher mGPS, and higher PI all predicted an increased mortality (*p* < 0.0001 for all), PLR did not.
(70)	Mortality	NLR, d-NLR, SII, and PNI all predicted mortality.
(81)	Mortality	NLR, d-NLR, and SII all predicted mortality in the univariate analysis; however, only SII was significant in the multivariate analysis.
(82)	Mortality	PNI independently predicted mortality in the multivariate analysis (AUC: 0.849). NLR and PLR significantly correlated in the univariate analysis.
(64)	Mortality, ICU admission, requiring invasive mechanical ventilation, and dialysis	Higher LCR on day 1 predicted an increased need for ICU admission and invasive mechanical ventilation. NLR could not predict any of the variables on day 1. Lower LCR on day 7 predicted increased mortality, while higher NLR correlated with requiring invasive mechanical ventilation and mortality.

SII, systemic immune-inflammation index; NLR, neutrophil to lymphocyte ratio; PLR, platelet to lymphocyte ratio; AUC, area under the curve; hsCAR, high-sensitivity C-reactive protein to albumin ratio; hsCPAR, high-sensitivity C-reactive protein to prealbumin ratio; PNI, prognostic nutritional index; LMR, lymphocyte to monocyte ratio; d-NLR, derived-NLR; AFR, albumin to fibrinogen ratio; CAR, C-reactive protein to albumin ratio; OR, odds ratio; LCR, lymphocyte to C-reactive protein ratio; SMD, standardized mean difference; mGPS, modified Glasgow prognostic score; PI, prognostic index.

We first compared disease severity reported by seven studies (**Table 1**) (30, 53, 59, 71–74). One of these was a meta-analysis of 20 studies, 19 on NLR and 5 on PLR, that found a correlation between higher NLR and PLR with disease severity. However, the mean standardized difference (SMD) for NLR was higher than PLR (2.80 versus 1.82) (59). Five of the six remaining studies found NLR superior to d-NLR, PLR, and LMR (30, 53, 71–74). The other study found d-NLR to be the only predictive marker in the univariate but not multivariate analysis among these four. NLR, PLR, and LMR did not correlate with disease severity (71).

NLR, PLR, and LMR could predict ICU admission in hospitalized patients; however, NLR (AUC: 0.861) could predict ICU admission better than PLR (AUC: 0.715) and LMR (AUC: 0.705) (75). Sun et al. concluded similarly and stated that only NLR correlated with the risk of ICU admission, while LMR and PLR did not (53). NLR, monocyte to lymphocyte ratio (MLR), and PLR could all predict progression to ARDS (65). Higher NLR could predict the need for mechanical ventilation (*p* = 0.003), but PLR was similar between patients requiring ventilation and those not (*p* = 0.41) (76).

NLR outperformed in prognosticating mortality compared with PLR (61, 66, 77, 78) and LMR (77). Three studies comparing NLR and d-NLR found that both could predict mortality (70, 77, 81).

While NLR had a greater predictive power for severe COVID-19 parameters, it did not seem to correlate with COVID-19 diagnosis compared with PLR and LMR. The studies defined correlating with COVID-19 diagnosis as having significantly different levels in COVID-19 positive and negative patients. Five studies compared NLR, PLR, and LMR based on their diagnostic ability (**Table 1**) (49, 65–68). Lissoni et al. specifically compared intubated COVID-19 patients and healthy individuals. They concluded that lower LMR, higher NLR, and higher PLR were observed in intubated patients with COVID-19 compared with healthy controls (49). Among the remaining four studies, NLR correlated better than PLR in two studies (66, 68) and worse in two others (65, 67). Only one of these studies contained the MLR, the inverted LMR variable (65). In this study, MLR had the highest AUC to differentiate healthy individuals from COVID-19 patients (0.892), followed by PLR (0.748) and NLR (0.722) (65). Overall, NLR was not superior to LMR and PLR in assisting diagnosis, but data are insufficient on this part to determine the best marker.

Only nine studies compared NLR to markers other than PLR, LMR, and d-NLR (61, 64, 68, 70, 71, 79–82) (**Table 2**). These studies provide valuable evidence but are not sufficient for an extensive assessment. Five of these studies measured NLR and SII (61, 68, 70, 71, 81), two of them without the possibility to compare the predictive ability of NLR and SII (61, 70). These two studies—one of them in cancer patients—found that NLR, d-NLR, SII, PNI, and mGPS could predict COVID-19 mortality, but it was not possible to determine the best predictive marker in these studies (61, 70). SII was superior to NLR in all the other three remaining studies comparing NLR and SII (68, 71, 81); one study related COVID-19 diagnosis (68), one for disease severity (71), and one for mortality (81). SII was also superior to d-NLR, MLR, and PLR in predicting mortality, with a small hazard ratio (HR = 1.0001, *p* = 0.029) (81).

Xue et al. concluded that hsCAR, hsCPAR, and PNI predicted COVID-19 severity in the multivariate analysis, while d-NLR and SII only correlated in the univariate analysis. NLR, LMR, PLR, and AFR could not predict severe COVID-19 (71). This study concluded the superiority of hsCAR, hsCPAR, and PNI. PNI was superior to NLR in predicting mortality and CAR in predicting disease severity, each in one study (79, 82).

In the study of Lagunas-Rangel, NLR was superior to LCR in predicting disease severity (SMD: NLR = 2.404, LCR = −0.912), although both were significant predictors (P: NLR = 0.001, LCR < 0.001) (80).

Altogether, these data suggest that some markers might produce more promising results than NLR, such as SII, PNI, CAR and hsCAR, and CPAR and hsCPAR. However, these markers are less studied compared with NLR. Although this section contained some of the comparison of other variables, a detailed discussion on each marker follows.

### 2.2 Platelet to Lymphocyte Ratio

PLR could help in diagnosing COVID-19. Four studies found a significant difference in the PLR of patients with positive SARS-CoV-2 compared to healthy individuals (49, 65–67), while only one concluded against this (68).

Two meta-analyses confirmed the effectiveness of higher PLR on predicting COVID-19 severity (59, 83). Higher PLR also correlated with an increased risk of severe disease in intubated COVID-19 patients (49). This ability to predict disease severity seemed to be optimal at its peak. PLR at peak could predict disease severity in the multivariate regression analysis; however, PLR at admission did not correlate with disease severity in the univariate analysis (84). Two studies studied the ability of PLR to predict ICU admission, and they produced conflicting results on this matter (53, 75).

Although PLR could predict disease severity in most of the studies, it was not able to predict mortality (61, 66, 77, 78), one specifically in cancer patients (61). Similarly, another study concluded that PLR is only slightly prognostic in predicting mortality in the univariate analysis among hospitalized patients (*p* < 0.001), but not in the multivariate analysis (*p* = 0.154) (82).

Owing to all the strengths of PLR in predicting various COVID-19-related parameters, it is a potentially suitable marker to triage COVID-19 patients. However, it seems to lack potentials to predict mortality and have a lower ability than NLR to predict several parameters.

### 2.3 Lymphocyte to Monocyte Ratio

Unlike neutrophil and monocyte count, a decrease in lymphocyte count correlated to multiorgan injury in COVID-19 patients (85). This was shown by Kazancioglu et al recently. However, in their study, monocyte count only correlated to SARS-CoV-2 infection but not severity (69). These studies provide the hypothetical bases for the prognostic value of LMR in COVID-19, as well as NLR and PLR.

Two studies compared the effectiveness of LMR in COVID-19 diagnosis, both finding a significant relationship between LMR and testing positive for SARS-CoV-2. In one of them, MLR (AUC: 0.892) was the best predictor compared with NLR and PLR, and 0.23 was declared the best MLR cutoff point (65). In another, significantly lower LMR was observed in intubated COVID-19 patients compared with healthy controls (49).

LMR did not correlate with disease severity in most studies (30, 71, 73, 74) except two (53, 72). Liu et al. showed that higher LMR could only significantly predict disease severity in the univariate analysis and also did not correlate with a longer hospital stay, higher hospital costs, and longer time to negative PCR (72). LMR could prognosticate progression to ARDS (65).

In two studies examining the ability to predict ICU admission, LMR did not correlate in one (53), and correlated but was inferior to NLR and PLR in the other (75). Data are limited regarding the ability of LMR to predict COVID-19 mortality; however, a study concluded the ineffectiveness of LMR in prognosticating this parameter (77).

LMR might have limited benefits in prognosticating COVID-19 (86), but its abilities seem to be lower than NLR and PLR, especially in predicting disease severity, ICU admission, and mortality. However, we encourage future studies to pursue the ability of LMR to recognize SARS-CoV-2 positive patients, as it demonstrated promises.

### 2.4 Lymphocyte to C-Reactive Protein Ratio

A limited number of studies examined this marker. The most important article on this is perhaps a meta-analysis on the role of LCR in predicting disease severity. They found a significantly lower LCR in patients with severe disease (SMD = −0.912, *p* < 0.001); however, it was less predictive compared with NLR (SMD = 2.404, *p* = 0.001). They based their results on five studies for each marker (80).

Higher LCR on day 1 predicted the need for ICU admission (adjusted OR: 3.1, *p* = 0.003) and invasive mechanical ventilation (adjusted OR: 2.5, *p* = 0.009), but could not predict in-hospital mortality (*p* = 0.60) and requiring dialysis (*p* = 0.44). Nevertheless, lower LCR on day 7 only correlated with an increased in-hospital mortality risk (adjusted OR: 0.1, 95% CI = 0.01–0.30, *p* < 0.0001) but not with the other factors (64).

### 2.5 Fibrinogen to Prealbumin Ratio and Albumin to Fibrinogen Ratio

Similar to most of the discussed markers, studies demonstrate a prognostic role for FPR and AFR in some cancers and other diseases with inflammatory pathophysiology (87–89). Nevertheless, only a few articles studied them to determine COVID-19 disease severity.

Lower AFR correlated with severe COVID-19 in univariate analysis (*p* < 0.0001), but not multivariate analysis (*p* = 0.079) (71). However, fibrinogen to albumin ratio (FAR), remained significant in predicting disease severity in the multivariate analysis in another study (HR = 4.058, 95% CI = 1.246–13.222, *p* = 0.020) (90).

### 2.6 C-Reactive Protein to Albumin Ratio and C-Reactive Protein to Prealbumin Ratio

CAR could predict disease severity in two studies (79, 91): one comparing it with NLR and finding a higher OR for CAR (OR = 17.652, *p* = 0.001) than NLR (OR = 1.512, *p* = 0.007) (79). However, the other study did not find as large an OR for CAR (1.264, *p* = 0.037) (91).

hsCAR and hsCPAR differ from their counterparts as they utilize high-sensitivity CRP (92). Xue et al. found that on-admission hsCAR, hsCPAR, and PNI significantly correlated with severe COVID-19 in the multivariate analysis among several other markers (NLR, LMR, FPR, PLR, SII, AFR). Furthermore, among these markers, only hsCPAR and hsCAR correlated with hospital stay length (71). In the Oh et al. study, hsCAR could also predict in-hospital mortality in adults older than 65 years of age after adjusting for confounders (92).

Taken together, CAR and CPAR seemed promising in predicting disease severity, mortality, and length of hospital stay in all the studies, although we only identified four studies. Furthermore, a small study hailed prealbumin and CRP as potential markers to effectively triage patients in the early stages, and prealbumin seemed to be more effective (93).

### 2.7 Glasgow Prognostic Score, Modified GPS, and High-Sensitivity mGPS

GPS constitutes two main serum components, CRP and albumin levels, both having a potential of 0 or 1 score. CRP >10 mg/L and albumin <3.5 mg/dl receive one point each, and the score classifies the patients into three total scores of 0, 1, or 2. mGPS does not allocate a score to hypoalbuminemia without a rise in CRP to above 10 mg/L. hs-mGPS provides a similar classification to mGPS with a lower CRP threshold (>3 mg/L) (94, 95). These markers also have proven roles in predicting various cancers (94, 95).

In a study of 397 patients with COVID-19, no deaths occurred among 40 patients with hs-mGPS score of 0, while 10/263 (3.80%) and 24/94 (25.53%) of patients scoring 1 and 2 died, respectively (70). Concurrently, Dettorre et al. found that mGPS was able to foretell the overall survival of hospitalized cancer patients infected with COVID-19 (11.4%, 30.4%, and 50.6% for mGPS = 0 to mGPS = 2, respectively; *p *< 0.0001) (61). These two studies found promising results for mGPS and hs-mGPS and set the grounds for future research to better identify its effectiveness.

### 2.8 Prognostic Index

PI is similar to the GPS, only differing in the WBC component. WBC >11,000/µl and CRP >10 mg/L contribute to this scoring system of 0 to 2 (22).

We could only find one study discussing this marker in COVID-19. In that study, PI was able to predict the survival of cancer patients infected with COVID-19 (9.1%, 40%, and 50%, for scores of 0 to 2), similar to NLR, PNI, and mGPs. PI seemed superior to PLR in the study. Patients categorized in the poor-risk group (PI = 2) had 23 days median overall survival, while patients with favorable scores did not reach the required follow-up duration (all *p*-values less than 0.0001) (61).

### 2.9 Prognostic Nutritional Index

Onodera et al. proposed PNI as an immune-nutritional risk score for malnourished cancer patients undergoing for gastrointestinal surgery, formulizing it as serum albumin concentration (g/L) + 0.005 × total lymphocyte count (per mm^3^ of peripheral blood) (96, 97). This marker later demonstrated its effectiveness in prognosticating several types of cancer (98).

Three studies investigated the relationship between COVID-19 mortality and PNI, all showing significant correlations (61, 70, 82). Two studies were on hospitalized COVID-19 patients (70, 82) and another on patients with cancer (61). PNI also predicted disease severity better than other markers in the study of Xue et al., alongside hsCAR, and hsCPAR. Together, they were put into a nomogram that could predict disease severity well (C-index = 0.873) (71).

PNI successfully predicted disease severity and mortality in all the four studies examining it; therefore, it can be a suitable candidate for follow-up studies.

### 2.10 Systemic Immune-Inflammation Index

SII is defined as platelet count × NLR (99). SII remained a reliable predictor in most of the conducted studies on COVID-19 so far. Usul et al. found its superior predictive ability than NLR and PLR in COVID-19 diagnosis, as its values were significantly different in SARS-CoV-2-positive and -negative individuals. The proposed SII for helping in the COVID-19 diagnosis was 479.1 (68).

Xue et al. studied the relationship between several markers and disease severity. They found that SII could significantly predict disease severity in the univariate but not the multivariate analysis, inferior to hsCAR, hsCPAR, and PNI, but still better than several markers, such as NLR (71).

Two studies studied SII in predicting mortality, both finding significant correlations (70, 81). One of them found that SII was the only significant marker in the multivariate analysis, superior to NLR and d-NLR, but with a slight hazard ratio (HR = 1.0001, *p* = 0.029) (81).

### 2.11 Interferon-Alpha Inducible Protein 27

IFI27 is a part of the innate immune system highly induced by interferon (IFN)-α (100). High expression of IFI27 may also induce cell proliferation and invasion and reduce apoptosis, making it a possible oncogene (100, 102).

Type I IFN deficiency can be a marker of severe COVID-19 (103). Some of the IFN-stimulated genes (ISGs) like IFI27 were upregulated in patients with COVID-19 (104–107) and later downregulated in the recovery process (108, 109). IFI27 was overexpressed in various cell lineages of SARS-CoV-2-infected patients compared with healthy controls (110). In another study, IFI27 demonstrated a higher than two-fold upregulation in A549 and normal human bronchial epithelial (NHBE) cells infected with SARS-CoV-2 (111). Shaath et al. analyzed the bronchoalveolar lavage of 10 individuals. IFI27 was among the genes upregulated in the peripheral blood mononuclear cells (PBMC) of severe and mild COVID-19 patients, compared with two healthy controls (112).

IFI27 might also help distinguish COVID-19 from other acute respiratory illnesses and some viral diseases such as Ebola, SARS, MERS, and H1N1, as SARS-CoV-2, even at low loads, induced IFI27 more than other viruses (113, 114).

There is a lack of robust clinical evidence concerning IFI27-related prognostic value for COVID-19; however, ISGs and, particularly, IFI27 seem to be interesting for conducting further studies.

## 3 Conclusion

**Figure 1:**
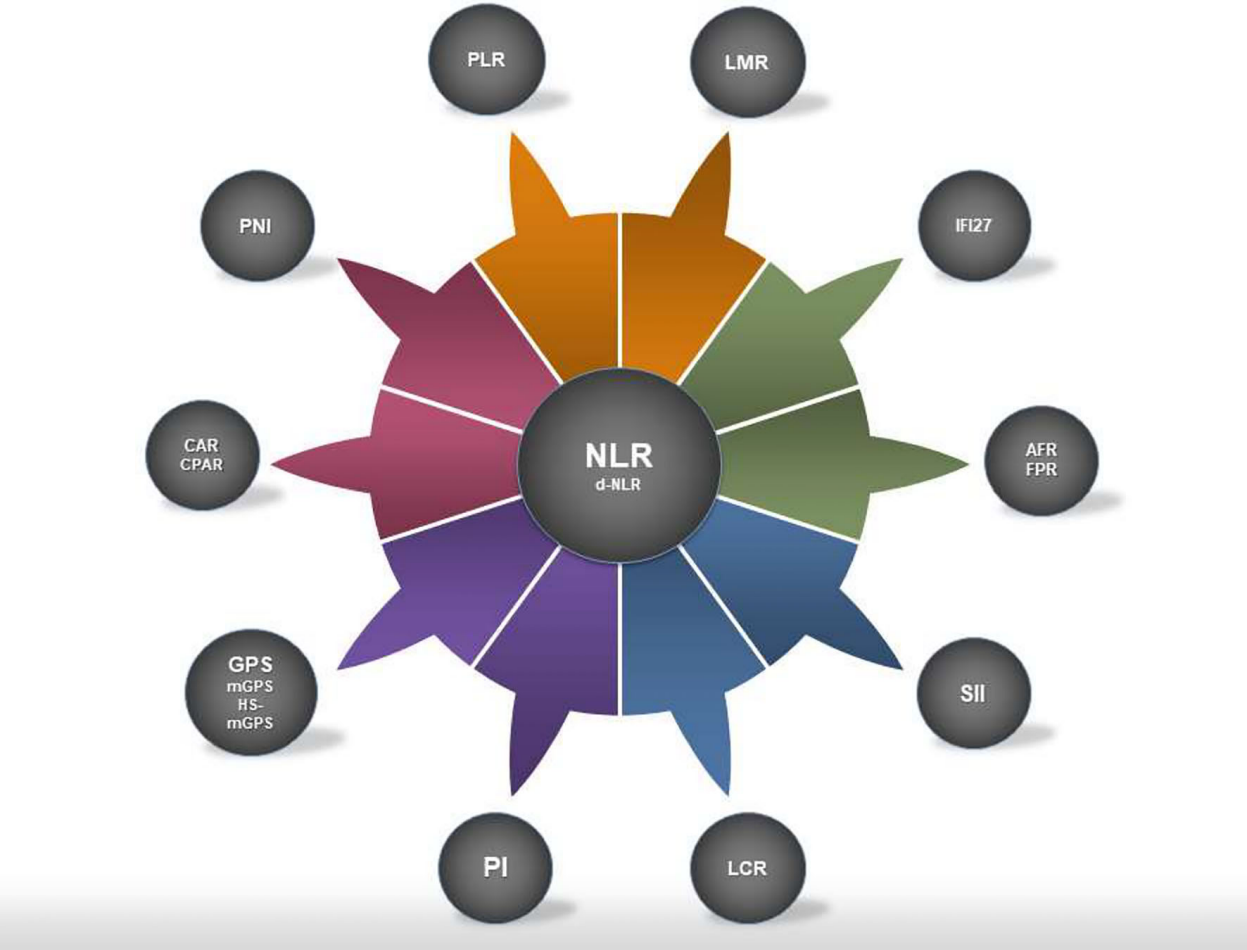
Systemic inflammation markers discussed in this study. NLR is illustrated in the middle as it was the most studied marker with strong prognosticating capabilities. However, some of the less studied markers reviewed in the study might have the potential to overtake the predictive value of NLR. NLR, neutrophil to lymphocyte ratio; d-NLR, derived NLR; PLR, platelet to lymphocyte ratio; LMR, lymphocyte to monocyte ratio; LCR, lymphocyte to C-reactive protein ratio; FPR, fibrinogen to prealbumin ratio; AFR, albumin to fibrinogen ratio; CAR, C-reactive protein to albumin ratio; CPAR, C-reactive protein to prealbumin ratio; GPS, Glasgow prognostic score; mGPS, modified GPS; hs-mGPS, high-sensitivity mGPS; PI, prognostic index; PNI, prognostic nutritional index; SII, systemic immune-inflammation index; IFI27, interferon-alpha inducible protein 27.

NLR seems to have the highest prognosticating potential among the biomarkers discussed in this study, because of its predictive value and availability for data across multiple studies, including meta-analyses. Therefore, this amplitude of evidence might increase its reliability to risk-stratify patients and help medical decision-making. Nevertheless, some other markers might also be promising, such as SII, PNI, CAR and hsCAR, and CPAR and hsCPAR, but other aspects of their prognostication need to be further studied (**Figure 1**). Careful comparisons require future meta-analyses.

Several studies discussed the markers for specific subgroups, such as patients with underlying diabetes or cancer. Many of these conditions might be inflammatory in nature, and they might hypothetically alter the effectiveness of some markers.

The need for risk-stratifying COVID-19 patients also encouraged some researchers to design new markers for this purpose that should be examined in studies, such as COVID-19 severity-Iraqi index (CSI) measured by MLR × lactate dehydrogenase (LDH)/upper normal LDH value (115). Another study hypothesized combining functional and nutritional indices with the well-known CURB-65 pneumonia severity index (116).

Some of the presented markers may only require a complete blood count with differentials, a cheap and straightforward test. The other markers also require routine and widely available laboratory tests. Therefore, stratifying the risks of patients using these methods has the potential of being widely available.

Some important pitfalls and limitations exist that future research need to address. First, studies need to estimate the cost-effectiveness of triaging the patients with these biomarkers, as almost all of them seemed to be useful to various degrees. Second, there is a lack of sufficient evidence for many of these biomarkers. Some of these markers have the potential to be better prognosticators than NLR, but need further studies to confirm their abilities and provide sufficient evidence. Third, we encourage researchers to hypothesize novel biomarkers best-fitted to COVID-19 pathophysiology and test their hypotheses to understand their effectiveness. We also encourage future research on specific subgroups with certain underlying conditions, as the most suitable biomakers for those groups might differ from the overall population. At last, various COVID-19 variants are showing different specific outcomes of morbidity and mortality (117). Therefore, we suggest future researchers to update the findings related to systemic inflammatory markers specifically for emerging variants.

## Author Contributions

AKa: conception, drafting of the initial manuscript, and revision of the final manuscript. PS: conception, drafting of the initial manuscript, and figure visualization. AKu: conception and careful revision of the manuscript draft. NR: conception and careful revision of the manuscript draft. All authors contributed to the article and approved the submitted version.

## Conflict of Interest

The authors declare that the research was conducted in the absence of any commercial or financial relationships that could be construed as a potential conflict of interest.

## Publisher’s Note

All claims expressed in this article are solely those of the authors and do not necessarily represent those of their affiliated organizations, or those of the publisher, the editors and the reviewers. Any product that may be evaluated in this article, or claim that may be made by its manufacturer, is not guaranteed or endorsed by the publisher.
